# Supplementary figures and images for: Daily actual evapotranspiration estimation of different land use types based on SEBAL model in the agro-pastoral ecotone of northwest China
Source: PLoS One. 2022 Mar 15;17(3):e0265138. doi: 10.1371/journal.pone.0265138 (PMC8923451; doi:10.1371/journal.pone.0265138)

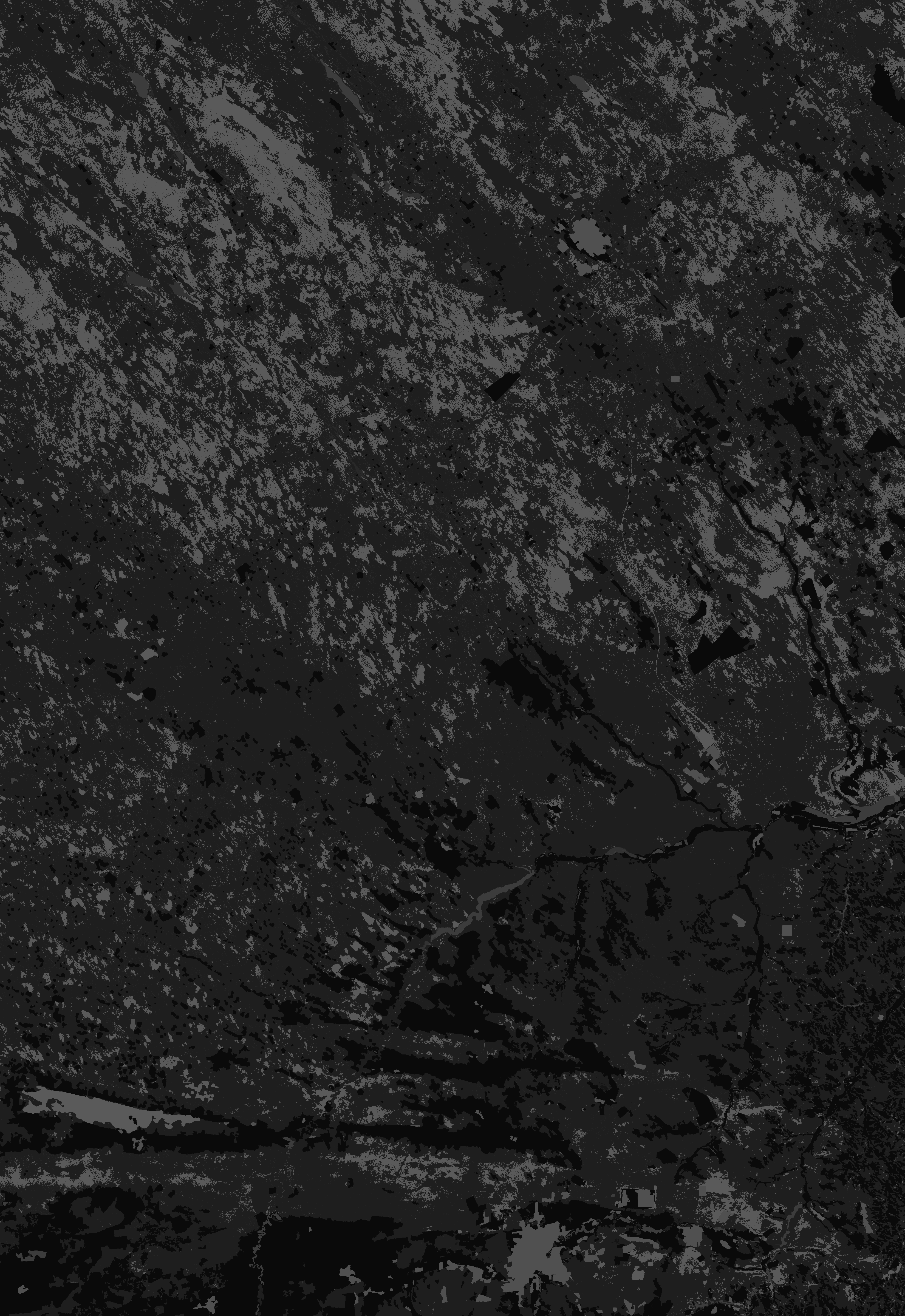

Supplement: S3 Dataset — (ZIP) [file pone.0265138.s003.zip › S3_Dataset/Land use types.tif]
