# Supplementary material for: Daily actual evapotranspiration estimation of different land use types based on SEBAL model in the agro-pastoral ecotone of northwest China
Source: PLoS One. 2022 Mar 15;17(3):e0265138. doi: 10.1371/journal.pone.0265138 (PMC8923451; doi:10.1371/journal.pone.0265138)
Supplement: Attachment — Submitted filename: PONE-D-21-26971R2.docx [file pone.0265138.s005.docx]

**PONE-D-21-26971R2**

Dear authors, I do appreciate your further efforts in producing an advanced version of your paper that takes into account my comments.

The paper has improved. I suggest accepting it, after the authors make the following necessary revision:

Normalized Difference Latent Heat (NDLI) index was proposed and proved as a reference for the assessment and monitoring water avalability. you are encouraged to use it for your validation.

Line 30 : “… coefficients of normalized vegetation indices…” Only NDVI was used for valiation as spectral indices…so used as singular noun

Please, improve the quality of the figure (as grip, scale…)
