# Supplementary material for: Daily actual evapotranspiration estimation of different land use types based on SEBAL model in the agro-pastoral ecotone of northwest China
Source: PLoS One. 2022 Mar 15;17(3):e0265138. doi: 10.1371/journal.pone.0265138 (PMC8923451; doi:10.1371/journal.pone.0265138)
Supplement: Attachment — Submitted filename: Response to Reviewers-R2.docx [file pone.0265138.s006.docx]

**Response to reviewers’ comments**

We would like to thank the academic editor and reviewers for their constructive and insightful comments and suggestions. We have revised the manuscript accordingly. To facilitate the reviewers to see the changes we made in response to their comments, the changes are marked in blue in the revised manuscript. Below we provide a point-to-point response to all the comments.

**Academic Editor**

**Question 1:** Please review your reference list to ensure that it is complete and correct. If you have cited papers that have been retracted, please include the rationale for doing so in the manuscript text, or remove these references and replace them with relevant current references. Any changes to the reference list should be mentioned in the rebuttal letter that accompanies your revised manuscript. If you need to cite a retracted article, indicate the article’s retracted status in the References list and also include a citation and full reference for the retraction notice.

**Response:** Our deepest gratitude goes to you for your careful work and thoughtful suggestions that have helped improve this paper substantially. We have revised the reference list to ensure that it is complete and correct. The changes of the reference list are marked in blue in the Revised Manuscript with Track Changes.

**Reviewer：**Dear authors, I do appreciate your further efforts in producing an advanced version of your paper that takes into account my comments.

The paper has improved. I suggest accepting it, after the authors make the following necessary revision:

**Questions 1:** Normalized Difference Latent Heat (NDLI) index was proposed and proved as a reference for the assessment and monitoring water availability. you are encouraged to use it for your validation.

**Response:** Thank you again for your support and affirmation of our work. Thank you very much for your suggestion. Since we have not used the Normalized Difference Latent Heat (NDLI) index in past research, this study has not used it for verification. Thank you for pointing us in new directions. And in future research work, we will introduce the Normalized Difference Latent Heat (NDLI) index to improve our research results. Thank you again for your affirmation and support of this research.

**Questions 2:** Line 30 : “… coefficients of normalized vegetation indices…” Only NDVI was used for valiation as spectral indices…so used as singular noun.

**Response:** We are extremely grateful to reviewer for pointing out this problem. We have fixed this question. “… coefficients of normalized vegetation indices…” modified to “… coefficients of normalized vegetation index…”. Thank you again for your support and affirmation of our work.

**Questions 3:** Please, improve the quality of the figure (as grip, scale…).

**Response:** Thanks for the suggestion. We have improved the quality of Figures 1, 2, 4, and 6, where Figure 1 has modified the scale, and Figures 2 and 4 have increased the resolution of the figures.
